# Factors associated with drug-related harms related to policing in Tijuana, Mexico

**DOI:** 10.1186/1477-7517-8-7

**Published:** 2011-04-08

**Authors:** Tyson Volkmann, Remedios Lozada, Christy M Anderson, Thomas L Patterson, Alicia Vera, Steffanie A Strathdee

**Affiliations:** 1 9500 Gilman Drive, MC-0507, La Jolla, California, 92093-0507, USA; 2 Patronato Pro-COMUSIDA, Zona Norte, Tijuana, Baja California, México

## Abstract

**Objective:**

To assess factors associated with drug-related harms related to policing among injection drug users (IDUs) in Tijuana, Mexico.

**Methods:**

IDUs who were over 18 years old and had injected drugs within the last six months were recruited via respondent-driven sampling and underwent questionnaires and testing for HIV (human immunodeficiency virus), syphilis and TB (tuberculosis). Random effects logistic regression was used to simultaneously model factors associated with five drug-related harms related to policing practices in the prior six months (i.e., police led them to rush injections; affected where they bought drugs; affected locations where they used drugs; feared that police will interfere with their drug use; receptive syringe sharing).

**Results:**

Of 727 IDUs, 85% were male; median age was 38 years. Within the last 6 months, 231 (32%) of IDUs reported that police had led them to rush injections, affected where they bought or used drugs or were very afraid police would interfere with their drug use, or shared syringes. Factors independently associated with drug-related harms related to policing within the last six months included: recent arrest, homelessness, higher frequencies of drug injection, use of methamphetamine, using the local needle exchange program and perceiving a decrease in the purity of at least one drug.

**Conclusions:**

IDUs who experienced drug-related harms related to policing were those who were most affected by other micro and macro influences in the physical risk environment. Police education programs are needed to ensure that policing practices do not exacerbate risky behaviors or discourage protective behaviors such as needle exchange program use, which undermines the right to health for people who inject drugs.

## Background

A growing body of literature about the risk of HIV (human immunodeficiency virus) and other blood-borne infections among injection drug users (IDUs) has focused on the influence of risk environments that shape individual behaviors [1-4]. These approaches stem from the understanding that IDUs' behaviors are a product of individuals' behaviors and their shared environments [2-4]. Drug users' right to health may be compromised by influences in their micro- and macro- risk environments [2-7]. Studies have identified macro-level factors, such as national drug possession enforcement policy [7] and repressive legal frameworks [5], and micro-level factors, such as discriminatory access to antiretroviral therapy [6] and problematic policing practices [3,5-13] as influences which may lead drug users to engage in risky behaviors, or prevent them from accessing potentially life-saving medicines and health services. Harm reduction strategies grounded in a rights-based approach should accordingly be focused on the interaction of the individual within structural risk environments at the micro- and macro-levels, which can be characterized in terms of the physical, social, economic, and policy influences [1-3].

Policing practices can exert strong influences on IDU behaviors in their micro-social environment [1]. Police practices that negatively affect the IDU risk environment are those that lead to increased harm for IDUs and an elevated risk of acquiring HIV and other blood borne infections [8-10,14]. The effects of police practices can be direct-for example, by confiscating syringes or causing IDUs to rush injections-or indirect, such as harassment that discourages IDUs from attending needle exchange or drug treatment programs [11,12]. Studies from a number of countries, including the US (United States), UK (United Kingdom), Australia, Russia, Ukraine, Mexico, and Canada have found that police pressure and policing practices are associated with high risk injection behaviors among IDUs [13,15,16]. In Canada, stopping, searching and detention of IDUs by police has been associated with both receptive and distributive syringe sharing [17,18]. Qualitative studies have examined the effects of direct and indirect policing practices on IDUs, emphasizing the adverse effects of these practices from a human rights perspective [19,20].

In the northern region of Mexico bordering the United States, HIV prevalence is rising among some IDU subgroups [9,21] and various policing practices have been shown to play a role in heightening IDUs' HIV risks. In Mexico, syringes can be purchased legally at pharmacies and can be carried without a prescription. However, as reported elsewhere [22], Mexican police often exert their own "law on the streets" that disregards "laws on the books" [23]. A qualitative study in two Mexico-US border cities, Tijuana and Ciudad Juarez showed that police violence and corruption affected their access to sterile syringes and the geographic locations where they injected drugs [24]. In both cities, nearly half of IDUs reported being arrested for carrying sterile or used syringes [24,25]. A more recent study in Tijuana found that 64% of IDUs reported ever being arrested simply for having track marks (i.e. injection stigmata) [8]. In both Tijuana and Ciudad Juarez, arrests for syringe possession were associated with a three-fold higher odds of receptive needle sharing [25]. Being arrested for syringe possession was also independently associated with shooting gallery use in both Tijuana and Ciudad Juarez [26], whereas being arrested for having track marks was independently associated with HIV infection among IDUs in Tijuana [8].

While these earlier studies have shown that police practices are associated with individual risk behaviors among IDUs, we are unaware of any prior research that has studied the effect of simultaneous drug-related harms related to policing within an IDU population, which is important because singular police actions could be associated with several simultaneous risk-taking behaviors [10]. We studied potential factors associated with the effects of policing practices on simultaneous drug-related harms among IDUs in Tijuana, Mexico in an effort to guide future intervention efforts.

For the purposes of this study, we refer to any policing practice that potentially causes harm to IDUs, including those mentioned above, as "drug-related harms related to policing." We operationalized drug-related harms related to policing as those where police actions caused IDUs to: 1) rush injections; 2) alter where they bought drugs; 3) alter geographic locations where they used drugs; 4) fear that police would interfere with their drug use; or 5) share syringes.

## Methods

### Setting

Tijuana, Mexico (population 1.5 million) is located south of San Diego, California in the northernmost part of western Mexico in the state of Baja California, and is a city facing the double threat of HIV and drug addiction. Up to 1 in 116 persons aged 15-49 in Tijuana was HIV-infected in 2006 [27]. In Baja California, 4.8% injected drugs, compared to Mexico overall, where 0.2% injected any drug in 2008, and rates of methamphetamine use in Baja were the highest in the country [27]. Approximately 10,000 people are thought to inject drugs in Tijuana [28].

Mexico has seen a surge in violence over the last four years-especially in the Mexico-US border region-due to conflicts among rival drug trafficking organizations [29]. This may have affected the policing environment by heightening awareness of drug activity and exerting periodic crackdowns on drug users across the region. Although Mexico recently enacted drug policy reform that deregulates possession of small, specified amounts of cocaine, heroin, methamphetamine and marijuana for personal use [30], the present study was conducted prior to this change in legislation and therefore presents baseline data from which changes in policing practices and their influence on IDUs can be compared in future investigations.

### Recruitment

Between 2006 and 2008, IDUs in Tijuana were recruited into a study of risk factors for HIV, syphilis, and tuberculosis infection using respondent driven sampling (RDS), as previously described [8]. Baseline eligibility criteria were: being 18 years or older; having injected drugs within the last month; being willing and able to provide informed consent; having no plans to leave the city over the next 18 months; and being able to speak English or Spanish. Study protocols were approved by both the Institutional Review board of the University of California, San Diego and The Ethics Board of the Tijuana General Hospital.

### Study instrument

At baseline, and semi-annually thereafter, participants were administered a survey that collected information on a variety of data, including sociodemographic variables (e.g., homelessness, place of birth), drug use and sexual behaviors, experiences with police and their perceptions and involvement in the local drug market. Drug use behaviors included: how often in the last six months they injected drugs alone or in combination, whether they engaged in receptive needle sharing, whether they used a local needle exchange program (NEP), and whether they had difficulty obtaining new syringes. Participants were also asked if they had been arrested ever or in the last six months, and if so, the reasons for the arrest(s).

Several questions were asked about their experiences and perceptions relating to police in the last six months. For example, participants were asked if police had caused them to rush an injection, whether police had affected the places where they bought drugs, whether police had affected the geographic locations where they used drugs, and to what extent participants were afraid that police would interfere with their drug use (e.g. "not afraid", "afraid", or "very afraid"). We asked about perceptions of the local drug market, such as whether the price, purity, and availability of specific drugs (i.e., heroin, cocaine, and methamphetamine) had increased, decreased or stayed the same in the last six months.

### Laboratory Testing

On whole blood obtained from venipuncture, rapid HIV testing was conducted to detect HIV antibodies using the Determine HIV test (Abbott Pharmaceuticals, Boston, MA). Positive samples were retested with an HIV-1 enzyme immunoassay and immunofluorescence assay. Syphilis antibodies were detected using the rapid plasma regain (RPR) test (Macro-Vue; Becton Dickinson, Cockeysville, MD); positive specimens were confirmed using the *Treponema pallidum *particle agglutination assay (TPPA; Fujirebio, Wilmington, DE). M. *tuberculosis *testing was done using an IGRA (QuantiFERON TB Gold In-Tube [QFT] assay; Cellestis Ltd., Carnegie, Victoria, Australia), a laboratory assay that utilizes ELISA to detect antibodies to specific target proteins similar to those released by M. *tuberculosis *[31]. All confirmatory testing took place at the San Diego County Health Department. Following pre- and post-test counseling, participants who tested positive for any of these infections were referred to the Tijuana municipal health clinic. Every attempt was made to engage all participants for follow-up through street outreach.

### Statistical analysis

Data for this study were obtained at the most recent follow-up visit, collected between October, 2007 and May, 2009. The outcome (drug-related harms related to policing) consisted of responses corresponding to the following five variables potentially related to adverse effects associated with policing practices: 1) police caused them to rush injections; 2) police affected where they bought drugs; 3) police affected geographic locations where they used drugs, 4) feared that police would arrest them or interfere with their drug use; and/or 5) receptive needle sharing. These outcome variables were chosen based on findings from previous studies conducted in earlier IDU studies in Tijuana [21,23-26], baseline analyses of this cohort [21,23], and pair-wise comparisons that investigated the interrelatedness of the outcomes and their suitability for simultaneous modeling. Based on earlier research from our group and others where IDUs reported that police pressure led them to resort to inject in shooting galleries [26], or in public spaces, we feel confident that IDU responses reflect a shift from safer to riskier spaces. While the wording of the question for receptive needle sharing was not framed as a sole response to policing practices, a previous analysis conducted among IDUs in Tijuana found that police confiscation of sterile syringes or used syringes was the strongest correlate of receptive needle sharing [25]. Phi coefficients and odds ratios for each pair of outcomes indicated positive correlations for all pairs [32].

Because participants' responses to these five questions were correlated, random-effects logistic regression was used to simultaneously model responses using generalized estimating equations (GEE) [33]. Each participant was characterized as a random effect in the regressions to account for the correlation of outcomes within participants, and each participant's RDS recruiter was considered as a random effect to account for correlation of participants who were recruited to the study by the same person, as previously described [8]. Univariable models were constructed to determine the simultaneous effect of police practices and other possible predictors on these five outcomes. Only variables which were associated with these outcomes at the univariable level (p ≤ 0.20) were considered for inclusion in the multivariable regression model. Multivariable models were constructed using a manual backward stepwise selection process, eliminating the least significant variables until all remaining variables were significant at p < 0.05.

## Results

Of the 1056 total participants enrolled in the study, data for 864 (81.8%) had been collected for the fourth study visit. Of these, 137 IDUs were excluded because they either had not injected drugs in the past six months (N = 134) or had missing values for any of the five drug-related harms related to policing or key covariates (N = 3). Compared to those who were included, participants excluded from the analysis were less likely to earn ≥3500 pesos per month (11.7% vs. 21.5%, p = 0.007); to have used marijuana or hashish (7.3% vs. 15.0%, p = 0.01), heroin (2.2% vs. 71.9%, p < 0.001), or methamphetamine (5.8% vs. 17.6%, p < 0.001); to have perceived a decrease in the purity of at least one drug during the six months prior to interview (0.7% vs. 6.2%, p = 0.006); to have been homeless (1.4% vs. 12.0%, p < 0.001); to have been arrested (8.0% vs. 25.2%, p < 0.001); to have been arrested for carrying used needles/syringes (0.0% vs. 2.9%, p = 0.04) or for having track marks (1.4% vs. 8.5%, p = 0.002); to have been beaten or sexually assaulted by the police (0.7%% vs. 6.2%, p = 0.006); to have had drugs planted, been asked for money, or had money taken by police (0.7% vs. 15.6%, p < 0.0001); and to have had belongings burned, been forced to leave their residence, asked for sexual favors, or have syringes taken by police (0.7% vs. 6.1%, p = 0.006). On the other hand, those excluded were more likely to have been in a prison or detention center, regardless of arrest (42.0% vs. 21.8%, p < 0.001).

Table 1 lists sociodemographic characteristics, drug-using behaviors and perceptions, and variables in the social and physical environment among IDUs in our sample. Participants were mostly male (85%), averaged 38 years of age, and nearly three quarters were single. Over the last six months, 79% had an average monthly income of less than 3500 pesos (approximately $300 dollars).

**Table 1:** Factors associated with drug-related harms, Tijuana, Mexico (Unadjusted ORs)

Covariate	*n *(%) (Total = 727)	Unadjusted OR	95% CI
**Sociodemographics**			
Female	112 (15.4%)	0.78	(0.54-1.14)
Age (Increase per 5 years)	38 (32 - 43)^‡^	0.90*	(0.83-0.97)
Married/common Law	196 (27.0%)	0.87	(0.65-1.17)
Earned ≥ 3500 pesos on average per month^**†**^	156 (21.5%)	3.08*	(2.40-3.96)
**Drug Using Behaviors**			
Used marijuana/hashish ^**†**^	109 (15.0%)	2.79*	(2.07-3.76)
Used heroin by itself ^**†**^	523 (71.9%)	1.08	(0.80-1.44)
Used methamphetamine by itself ^**†**^	129 (17.7%)	2.57*	(1.95-3.38)
Injected drugs more than once per day^**†**^	432 (59.4%)	3.10*	(2.21 - 4.37)
**Physical Macroenvironment**			
Perceived decrease in purity of at least one drug^**†**^	46 (6.3%)	3.44*	(2.17-5.43)
Deported from the United States	263 (36.4%)	1.00	(0.76-1.32)
**Physical Microenvironment**			
Homeless^**†**^	88 (12.1%)	1.72*	(1.23-2.40)
Born outside Baja, California	475 (65.6%)	0.99	(0.75-1.30)
Spent time in jail, prison, or detention center^**†**^	159 (21.9%)	1.88*	(1.39-2.54)
**Social Microenvironment**			
Traded sex^**†**^	16 (4.4%)	2.42*	(1.33-4.40)
Police affected access to sterile needles^**†**^	7 (1.0%)	1.14	(0.44-2.93)
**Social Microenvironment**			
Perceived an increase in price of at least one drug^†^	4 (0.6%)	3.06*	(1.27-7.35)
**Political Microenvironment**			
Used local needle exchange program^**†**^	452 (62.2%)	2.74*	(1.94-3.89)
Hard or very hard perceived ease to get new, unused syringes^**†**^	6 (0.8%)	3.80*	(1.68-8.57)
**Experience with Police Practices**			
Arrested^**†**^	183 (25.2%)	2.76*	(2.12-3.59)
Arrested for carrying drugs^**†**^	34 (4.7%)	3.31*	(2.06-5.33)
Arrested for carrying sterile needles/syringes^**†**^	16 (2.2%)	3.69*	(1.84-7.42)
Arrested for carrying used needles/syringes^**†**^	21 (2.9%)	3.20*	(1.70-6.01)
Arrested for having track marks^**†**^	62 (8.5%)	2.40*	(1.62-3.58)
Beaten or sexually assaulted by police (police violence)^**†**^	45 (6.2%)	1.97*	(1.21-3.20)
Had drugs planted, was asked for money, or had money taken by police (police corruption) ^**†**^	114 (15.7%)	2.38*	(1.72-3.28)
Had belongings burned, forced to leave, asked for sexual favors, or had syringe taken by police (police coercion) ^**†**^	45 (6.2%)	3.10*	(1.96-4.91)
**Prevalence of Infections**			
Positive for HIV	37 (5.1%)	0.68	(0.38-1.21)
Active syphilis titer ≥1:8	36 (5.1%)	0.33*	(0.14-0.78)
Positive for TB Infection	539 (76.6%)	0.91	(0.67-1.25)

* *p *< 0.05

^†^Past 6 months

^‡^Median (IQR)

In terms of drug use characteristics, most (72%) used heroin by itself in last six months, and 59% injected drugs more than once per day. Over half (62%) reported using the local NEP within the past six months. A minority (6%) reported perceiving a decrease in the purity of at least one drug in the last six months; 0.6% perceived an increase in the price of at least one drug; 1% found it difficult to get new syringes and 12% described themselves as homeless.

In terms of experiences with police, 25% reported having been arrested for any reason since their last interview. In addition, 16% reported being victims of police corruption, defined by having drugs planted on them, being asked for money, or having money taken by police; In terms of health, 77% were positive for tuberculosis infection, 5.0% for active syphilis, and 5.1% tested HIV-positive.

In terms of the drug-related harms potentially related to policing, 4% reported that police had led them to rush injections; 1% said police affected where they bought drugs; 2% responded that police affected where they used drugs; 4% were very afraid that police would interfere with drug use; and 28% reported receptive needle sharing. Nearly one-third (32%) reported any of these five drug-related harms.

### Univariable Analysis

Factors associated with drug-related harms related to policing are shown in Table 1. Older IDUs were significantly less likely to report drug-related harms (Odds Ratio (OR) = 0.90 per 5 years, 95% CI: 0.83 - 0.97), and IDUs who reported earning at least 3500 pesos per month were more likely to experience drug-related harms (OR = 3.08, 95% CI: 2.40 - 3.96). IDUs who used marijuana were significantly more likely to report drug-related harms (OR = 2.79, 95% CI: 2.07 - 3.76), as were IDUs who used methamphetamine (OR = 2.57, 95% CI: 1.95 - 3.38), injected drugs more than once per day (OR = 3.10, 95% CI: 2.21 - 4.37), or used the NEP (OR = 2.74, 95% CI: 1.94 - 3.89).

IDUs who were homeless were significantly more likely to report drug-related harms related to policing (OR = 1.72, 95% CI: 1.23 - 2.40). IDUs who had been arrested in the past six months were significantly more likely to report drug-related harms (OR = 2.76, 95% CI: 2.12 - 3.59), as were IDUs who spent time in jail in the past six months (OR = 1.88, 95% CI: 1.29 - 2.54). IDUs who reported being victims of police violence (OR = 1.97, 95% CI: 1.21 - 3.20), police corruption (OR = 2.38, 95% CI: 1.72 - 3.28), or police coercion (OR = 3.10, 95% CI: 1.96 - 4.91) were also more likely to report drug-related harms.

### Multivariable Analysis

Variables that remained independently associated with drug-related harms related to policing are displayed in Table 2, with the recall period for all variables being the last six months. These were being arrested (adjusted odds ratio (aOR): 1.93; 95% CI = 1.46-2.55); having been homeless (aOR: 1.58; 95% CI = 1.12-2.23); injecting drugs more than once per day (aOR: 2.01; 95% CI = 1.42-2.85); using methamphetamine (aOR: 1.92; 95% CI = 1.43-2.57); using a needle exchange program (aOR: 1.91; 95% CI = 1.34-2.71); and perceiving a decrease in the purity of at least one drug (aOR: 2.09; 95% CI = 1.25-3.50). Consideration of other variables significant in univariable analyses did not appreciably alter these results.

**Table 2:** Factors independently associated with drug-related harms, Tijuana, Mexico --odds ratios for fixed effects (n = 727)

Variable	Adjusted OR	95% CI
**Social microenvironment**		
Arrested^†^	1.93*	(1.46-2.55)
**Physical microenvironment**		
Homelessness^†^	1.58*	(1.12-2.23)
**Drug Using Behaviors**		
Injected drugs more than once per day^†^	2.01*	(1.42-2.85)
Used methamphetamine by itself^†^	1.92*	(1.43-2.57)
**Political Microenvironment**		
Used local needle exchange program	1.91*	(1.34-2.71)
**Physical macroenvironment**		
Perceived a decrease in purity of at least one drug^†^	2.09*	(1.25-3.50)

* *p *< 0.05

^†^Past 6 months

## Discussion

In this study of IDUs in Tijuana, Mexico, those who were most adversely affected by drug-related harms related to policing in the prior six months were those who were most affected by other influences in the micro-physical environment (i.e., homelessness) and macro-physical environment (i.e., perceived changes in the local retail drug market). They were also more likely to have used the local NEP, to be more frequent injectors, and to be more likely to use methamphetamine. While it is imperative that structural interventions are developed to reduce problematic policing behaviors using a rights-based approach, this analysis helps to identify a subgroup of IDUs who appear to be most vulnerable to policing practices and who are in need of additional supports.

Our finding that recent homelessness was independently associated with drug-related harms related to policing supports those from a study in Vancouver, Canada [17], which found that IDUs most affected by policing practices may be at risk for other adverse outcomes, such as homelessness and drug-related harms. These IDUs were more likely to frequently use crack cocaine, to require help injecting, and were more likely to engage in distributive syringe sharing. Being homeless may be associated with a more disheveled appearance and a tendency to inject in public spaces, rendering these IDUs more identifiable to police. In Tijuana, homeless IDUs often live in the Tijuana River canal, where they are frequently targeted during police sweeps. Although syringe possession is legal in Mexico, a recent Tijuana study showed that homelessness was associated with pharmacists' refusal to sell syringes to IDUs, with some pharmacists remarking on their unkempt appearance [34]. At baseline in our study, 64% of Tijuana IDUs had been arrested for having 'track marks' [8], which suggests that IDUs' who are more easily identified as IDUs may be more vulnerable to policing and stigmatization. A qualitative study of IDUs from Russia concluded that internalized stigma within the police force is a product of structural violence that violates the right to health [19]. In New York City, illicit drug users who reported stigma, measured by alienation and discrimination, were more likely to have poorer mental and physical health [35]. These findings suggest that programs that provide temporary shelter and housing, access to toiletries and clothing to IDUs may reduce their vulnerability to adverse policing practices and other forms of stigmatization.

In our study, IDUs who reported drug-related harms related to policing were also those most affected by influences in the macro-physical environment; in this case, perceived changes in the retail drug market. Specifically, IDUs who reported perceiving a decrease in the purity of at least one drug during the prior six months were more than twice as likely to report drug-related harms related to policing. Because these IDUs were also more frequent injectors, they may be more dependent on drugs, less financially secure, and subject to the volatility of the drug market. The socio-demographic profile and drug use patterns of IDUs in Tijuana who reported drug-related harms consisted of younger, more frequent injectors who were more likely to use methamphetamine. Methamphetamine in particular has been associated with chaotic drug use patterns [36], frequent injection [37], and elevated levels of violence [38], which could draw greater attention from police.

We found that Tijuana IDUs who used the local NEP were almost twice as likely to be to report drug-related harms related to policing. One interpretation is that police may target IDUs using the NEP to meet arrest quotas, which undermines harm reduction efforts. A study in the United States showed that police presence and arrest was associated with a decrease in NEP utilization by IDUs [39]. IDUs who depend on NEPs and who are targeted by police at NEP locations may have difficulty identifying other sources of sterile injection equipment, and may engage in unsafe behaviors such as sharing or renting/buying syringes at shooting galleries. Alternative interpretations could explain our results. For example, homeless and/or methamphetamine injecting IDUs may be frequent injectors who need syringes more frequently. If they are unable to purchase syringes at pharmacies due to their appearance, they may be more likely to use NEPs, where they are more visible to police, and are more likely to be arrested and report drug-related harms related to policing. Without knowing the geographic location of arrest, it is impossible disregard this potential explanation. Prospective studies incorporating mixed methods research should be conducted to explore the context and elucidate potential causal pathways for arrest of IDUs. Regardless of the mechanism, ongoing efforts are needed to educate police at multiple levels to ensure that policing practices do not undermine harm reduction efforts. In Tijuana, this has been particularly challenging due to the presence of municipal, state and federal police, and sometimes the army, in response to drug-related violence.

This analysis was subject to some limitations. The cross-sectional design precludes us from drawing causal inferences. Low power may explain why HIV, TB, and syphilis associations were not significantly associated with our outcome variables. Additionally, our group and others have previously noted that the impact of policing practices on biological outcomes such as HIV infection is likely to be mediated by other variables, and that these associations might not be linear [3,40]. Since this sample was drawn from an ongoing cohort study, responses may be subject to some degree of socially desirable responding, which can lead to bias. Since this analysis was conducted based on data collected at a later follow-up visit, our analysis excluded some lower risk IDUs and over-represented those who were more severely affected by adverse policing practices, which may have led some associations may have been over-estimated. On the other hand, IDUs who were excluded were also more likely to have been previously incarcerated, which could suggest that some associations are under-estimates. Although 90% of the IDUs examined at baseline attended at least one follow-up visit, highly mobile persons were not eligible for this study, which might have led us to underestimate some associations.IDU behaviors and other influences in the risk environment may have changed over time, which may have influenced our results. The small subgroup of IDUs who were sex workers was relatively small and low power may have precluded observing a significant association related to the sex trade. Finally, our results are not generalizable to IDUs outside Tijuana.

A growing body of literature suggests that IDU behaviors are a product of individuals and their shared structural environment [4]. This shifts the onus of responsibility for change away from people who inject drugs, placing greater emphasis on policymakers and governments [3,4]. Our findings extend our earlier research from Tijuana and support the growing body of literature that identifies policing practices as an important driver of drug-related harms [8,10]. These findings collectively underscore the importance of interventions that target problematic policing practices. Police trainings should be offered that include education on the medical models of addiction and the role of appropriate public health responses, which include harm reduction to reduce drug-related harms to individuals and communities [41]. Police education programs focused on harm reduction are currently being developed for police cadets in Tijuana and Ciudad Juarez with the hopes that these initiatives will promote a climate of respect for drug users as people, and respect the role of NEPs and drug treatment in prevention and recovery. In the meantime, our results help identify a subgroup of IDUs who are more adversely affected by police practices who are in need of supportive community-based programs. Basic services such as temporary housing, clothing and bathing facilities may help decrease stigmatization and vulnerability to police, which can ultimately protect drug users' right to health.

## List of abbreviations

IDU: Injection drug user; NEP: Needle exchange program; RDS: Respondent-driven sampling; TB: Tuberculosis; HIV: Human immunodeficiency virus; US: United States; UK: United Kingdom; RPR: Rapid plasma regain; OR: Odds ratio; aOR: adjusted odds ratio

## Competing interests

The authors declare that they have no competing interests.

## Authors' contributions

TV contributed to initial analyses, manuscript development, and editing. RL contributed to study design and manuscript editing, and served as a liaison to our Mexican counterparts. CA ran analyses, wrote the methods and results section, and contributed to editing. TP contributed to study design, analyses, modeling, and editing. AV contributed via data collection and manuscript drafting. SS was PI and contributed heavily to direction of analyses, manuscript drafting, and editing. All authors read and approved the final manuscript.
